# Early treatment with infliximab in patients with juvenile idiopathic arthritis

**DOI:** 10.1186/1546-0096-9-S1-P113

**Published:** 2011-09-14

**Authors:** TV Sleptsova, EI Alexeeva, SI Valieva, TM Bzarova, KB Isayeva, RV Denisova, EV Mitenko

## Background

Treatment of patients with JIA with DMARDs is started immediately after diagnosis, resulting in more effective suppression of disease activity. TNF blockers are recommended in cases of active JIA after the unsuccessful use of DMARDs. The exact role of these agents in the treatment of early-stage JIA is unknown.

## Objective

To evaluate the efficacy of infliximab in patients (n=100) with early and long-standing JIA.

## Methods

100 (60 with early and 40 with long-standing JIA) patients who didn’t respond to DMARDs received infliximab 6-7 mg/kg q8wks. Evaluation of efficacy included 30%, 50% and 70% improvement by the ACR-pedi criteria and remission.

## Results

At 54 week 100% and 87.5% patients with early and long-standing JIA respectively achieved at least 50% response. After 2 years ACR-Pedi 70/90 response to infliximab was recorded in all patients with early JIA and 100%/88.9% of patients with long-standing JIA respectively. At weeks 54 following infliximab treatment, 89% and 60% of patients with early and long-standing JIA achieved remission. At the end of the second year, remission was reported in 97% of children with early JIA and 72% of patients in the second group. 34% of the patients discontinued due to an adverse event, mainly lack of efficacy (23 patients) and hypersensitivity reactions (11 patients).

## Conclusions

This 2-years study suggest using IFX as initial treatment for patients with recent onset JIA is more effective than reserving it for patients with long-standing JIA. No difference between groups in adverse events and secondary inefficacy were observed.

